# COVID-19 Contact Tracing Solutions for Mass Gatherings

**DOI:** 10.1017/dmp.2020.241

**Published:** 2020-07-14

**Authors:** Benjamin J. Ryan, Damon Coppola, James Williams, Raymond Swienton

**Affiliations:** Baylor University, Waco, Texas; Shoreline Risk, LLC, Virginia; Disaster Speak, PTY, Queensland, Australia; University of Texas Southwestern, Dallas, Texas

**Keywords:** contact tracing, COVID-19, environmental health, mass gathering

## Abstract

Mass gatherings and high-density activities, such as sporting events, conventions, and theme parks, are consistently included among highest-risk activities given the increased potential for widespread coronavirus disease 2019 (COVID-19) transmission. A more balanced risk management approach is required because absolute suppression of risk is unrealistic in all facets of life. Contact tracing remains a limiting factor in achieving such a balance. The use of Bluetooth or pairing devices is proposed to address this challenge. This simple approach, when applied in a manner that satisfies privacy and trust concerns, would allow high-risk encounters to be quickly identified, namely those where participants have spent 15 minutes or more within 6 ft of each other per current guidelines. If an attendee later tests positive for COVID-19 and tracing is required, the event organizer can provide a limited list of potential close contacts rather than an exhaustive list of all attendees. Contact tracers can, therefore, limit efforts to this concise group rather than needing to contact thousands of people or conduct mass media communications. Such a system, if institutionalized, supports risk assurance and safety measures for businesses by demonstrating a commitment to staff, customer protection, and ensuring high-risk encounters are logged, reinforcing longer-term societal pandemic resilience.

The coronavirus disease 2019 (COVID-19), caused by the severe acute respiratory syndrome coronavirus-2 (SARS-CoV-2) virus, spreads primarily person to person through respiratory droplets and saliva when an infected person sneezes, coughs, or talks.^1^ Transmission also occurs when people touch a contaminated surface or object and subsequently touch their mouth, nose, or eyes.^2^ The bulk of transmissions occur when an uninfected individual remains in prolonged (15 min or more) close contact (6 ft or less) with an infected person.^2,3^ The extremely-high transmissibility of SARS-CoV-2 is similar to what has been observed with the 4 “common cold” coronaviruses that have caused mild to moderate respiratory tract illnesses across the world for decades.^4,5^ Despite the relative simplicity of measures required to reduce interpersonal transmission, namely handwashing, physical distancing measures, and self-isolation when ill, public adherence is generally insufficient on account of social norms, habits, and active resistance.^6^

COVID-19 poses the greatest health risk to those over 65 or who have serious underlying health conditions. In the United States, 94% of those who have died from COVID-19 had at least 1 underlying condition,^6^ while in Italy 99% of fatalities were qualified as such.^7^ In the United States, nearly half of the population regardless of age have at least 1 chronic disease.^8^ The most common comorbid diseases or conditions include: diabetes, heart disease, liver disease, moderate to severe asthma, chronic lung disease, undergoing dialysis (chronic kidney disease), severe obesity (body mass index of 40 or higher), and reduced immunocompetence (undergoing cancer treatment, and poorly controlled AIDS or HIV infection).^9^

A person is considered COVID-19 exposed if they have: a household member or intimate partner with the disease, provided care to an infected person without using infection control prevention, or spent 15 min or longer in close contact (less than 6 ft) with an infected person.^10^ This close contact criterion is consistent with the Center for Disease Control and Prevention (CDC) Interim U.S. Guidance for Risk Assessment and Work Restrictions for Healthcare Personnel with Potential Exposure to COVID-19. The timeframe currently considered for close contact with an infected person begins 48 h before symptoms present or 10 d before positive specimen collection for asymptomatic clients, and lasts for 10 d after symptoms onset or 3 d after fevers have ceased.^11^ Self-isolation of exposed persons is recommended for 14 d following exposure.^12^ Without self-isolation, exposed individuals who become infected can continue to pass the disease to others, leading to a chain of infection.

Contact tracing, wherein potentially infected individuals are contacted and informed of their need to self-isolate, is the best available tool for ensuring infection rates remain suppressed.^13^ If decision-makers can be assured that exposures are traceable and exposed individuals can be informed of their need to self-isolate, the risk associated with permitting mass gatherings, such as theme parks, sporting events, and conventions is significantly reduced. Contact tracing capabilities, however, remain inadequate due to the number of trained tracers needed to contact a growing number of potential exposures. The potential of significantly increased tracing requirements posed by mass gatherings, where a large number of potential contacts can occur in a short period of time, is the basis of extended moratoria on such events or activities. However, in reality the number of meaningful contacts (15 min within 6 ft of infectious persons) is likely to be unknown. The challenge is in identifying who among the large number of attendees present have met the standards of a high-risk contact. To provide a path forward, this study outlines mass gathering reopening considerations and highlights how event specific contact tracing capabilities can support health department activities while also enabling societal pandemic resilience.

## Mass Gathering Reopening Considerations

As communities begin initiating incremental reopening plans, mass gatherings and high-density activities, such as sporting events, conventions, concerts, festivals, and theme parks, are consistently deferred given the increased potential for widespread COVID-19 transmission.^14^ These events and activities represent a significant source of both revenues and employment for many communities and businesses, and, therefore, special consideration is merited to identify alternative means of low-risk resumption. Absolute suppression of risk is unrealistic in all facets of life, but a more balanced risk management approach is suggested to enable safer resumption of activities that offer significant social and economic benefits.^15^ While theme parks, sporting events, conventions, and festivals cannot operate exactly as they did before the current crisis, with effective risk mitigation measures in place a modified resumption is possible.

Regaining public trust is an important consideration in the reestablishment of future mass-gathering events. Social activity and community reintegration into entertainment venues, sports stadiums, convention centers, schools, universities, and houses of worship will require establishing a new norm of safety, risk monitoring, and effective personal communication with attendees. The utility and methodology of proper scientifically valid contact tracing is an important factor in establishing public trust in this new norm.^16^ Public participation will be impacted by the perception of personal empowerment through effective risk communication and monitoring compliance.^17,18^

Incremental opening is already occurring as guided by several national and state-driven approaches. Two examples include the White House Opening Up America Again framework and the Opening the State of Texas plan.^19,20^ These plans, as well as many others throughout the country, are incrementally allowing restaurants, service-based operations, and other nonessential venues to reopen at reduced capacity (for example, 25-50% initially and increasing to steadily larger capacities as transmission control is sustained).^19^ Using such measures as reference, mass gatherings could also incrementally resume with special infection control measures in place and in conjunction with the ongoing effectiveness of broader public health strategies (Table 1)

**TABLE 1:** Phased Approach to Incrementally Reopening Mass Gatherings

Phase	Capacity	Required Measures	Phase Considerations – Entry, Up, or Down
Phase 1 Phase 2 Phase 3	25% 50% 75%	Sanitation Hygiene Physical distancing Contact tracing capability	County and/or state identify commencement phase. Trajectory of documented cases or positive tests within a 14-d period, hospital capacity and contact tracing availability. Evaluate event outcomes from a COVID-19 perspective, including compliance with measures.
Reopen at full capacity			

The criteria for increasing the capacity of mass gatherings (for example, from 25% to 50%) could align with the phase considerations but likewise incorporate event-specific considerations drawn from a COVID-19 risk evaluation perspective to include compliance with required measures by facility staff and patrons. A key measure to consider is a solution that addresses 1 of the primary barriers for mass gatherings, tracking of intimate/close contacts between strangers.^21^ If there were no issues identified the mass gathering capacities could then move to phase 2 and so on. Applying this type of methodology provides a systematic mechanism to safely open and control mass gathering sizes as the COVID-19 pandemic continues.

To begin resumption of mass gatherings, the strategy adopted must mirror the local public health context as is exemplified in Table 1, rather than following any national directives. The COVID-19 pandemic has exhibited a different impact between states and jurisdictions due to resident mobility, population density, locally imposed protection measures, demographics, and capacity of the medical system, and these differences will likewise impact the risk posed by mass gatherings.^21^ Use of sanitation is the first and most effective line of defense, and might include an event mandate on temperature checks on entry (or of all employees), use of face masks in certain situations, increased availability of handwashing stations, regular cleaning of common-use equipment (eg, railings), propping of bathroom doors, and elimination of common access items (eg, condiment stations at food concessions).

Careful consideration must also be given to the type and setting of activities considered. Some venues enable safer participation based on the flow of traffic, room to spread out, points of congregation, and other factors. For instance, a sporting event will result in sustained contact with a very small number of patrons in nearby seats (which can be staggered to increase distancing) or in concession lines (which can also be staggered), while a convention, festival, or theme park may have increased opportunities for comingling that require increased use of effective transmission control mechanisms (for example, assigned times to access a ride).

The specific guidance for sanitation, hygiene, and physical distancing provided by the CDC could be enforced and monitored at the county and state by the environmental health workforce.^22^ The role of this profession has rapidly evolved over recent years to include emergencies to protect the public from exposures to environmental hazards, disasters, and disease outbreaks.^23^ In recognition, the profession has been formally recognized in the Pandemic and All-Hazards Preparedness and Advancing Innovation Act of 2019. In this situation, the activities would build on existing skills and tasks. This could include, for example, ad-hoc inspections of sanitation, hygiene, and physical distancing standards at mass gatherings and liaising with event organizers to identify and mitigate potential transmission risks. This could be rapidly and easily achieved as the environmental health workforce are generally government employees who have the mandate and authorities necessary to enforce health directives. Also, they are often experienced in working with business and the community in stressful and complex situations.^22^

These events are not dissimilar from many of the permitted activities in terms of the person to person risk. Certain activities are also more heavily impacted by physical distancing, particularly conventions, concerts, sporting events, and festivals. However, just as many people may go into a concert as go into a supermarket or big box store across a given day. If this is the case, contact tracing system using Bluetooth or similar pairing device would be able to determine if this theory is correct by identifying the number of people a person is exposed to for more than 15 min (within 6 ft). The result could be an extremely low number of people (with most contacts being mere seconds) as would occur during any hike along a crowded trail or at a supermarket. By applying specific measures to high-density events, such as a festival, many of the factors that contribute to risk can be counteracted, thereby enabling such events without the same concern of ultimately creating new outbreaks. This leads us to the next section, which is that enhanced tracing capabilities are among the greatest resources in the effort to make these events possible.

## Contact Tracing Capabilities for Mass Gatherings

A critical component of community and country reopening strategies is contact tracing. This requirement is of particular significance in the context of mass gatherings where a large number of contacts occur in a short period of time. Tracing breaks the cycle of exponential growth typified in epidemic and pandemic crises by enabling identification and containment of new infections before the newly infected person begins transmitting the disease to others. The Johns Hopkins Bloomberg School of Public Health and Association of State and Territorial Health Officials (ASTHO) report A National Plan to Enable Comprehensive COVID-19 Case Finding and Contact Tracing in the US explains that a national initiative combining increased testing, enhanced public health workforce capacities, and adoption of new technologies for case identification and contact tracing is needed in each state.^24^

As testing capabilities increase, the demand for contact tracing increases in tandem where infections exist given the number of contacts rises exponentially with each positive test. This resource-intensive process includes identifying, assessing, and managing people who have been exposed to COVID-19 (or any other disease) to prevent onward transmission.^25^ Contact tracing has been routinely used for diseases such as tuberculosis, measles, sexually transmitted infections, and Ebola.^24^ It was effectively used in Shenzen, China, to help control the spread of COVID-19 by reducing the reproduction number to 0.4 (if the number is less than 1 the outbreak dies).^26^ In comparison, the World Health Organization suggests the reproductive number of COVID-19 without intervention is 1.4 to 2.5.^27^

In light of the contact tracing requirement, mass gatherings pose several key challenges. From a capacity standpoint alone, the scope of potential contacts of any infected person could feasibly include every person present at that event. Whereas a typical infection might result in 3.2 prolonged contacts (based on a study in China where 391 COVID-19 patients had 1286 close contacts^26^), sporting events, festivals, and concerts may have tens of thousands of attendees or more, any of whom may have come into contact with another attendee who is later found to have been infectious at the time of the event. Another problem arises from the ability to contact individuals who have attended such events. There is also a high risk of inaccuracy when people are asked to recall their contacts, especially as many will not have realized such contacts occurred in the setting of a mass gathering event (for example, while waiting in line).

While mass application of contact tracing technologies remains unrealistic due to privacy and feasibility challenges, the same does not necessarily apply to many mass gatherings. Audiences and attendees are already accustomed to providing identification upon entry, using bracelets, hand-stamps, or lanyards to verify entry, and complying with facility rules and regulations. Through the use of emerging yet inexpensive communications technology, many if not all of the major challenges to tracing at mass gatherings can be overcome. For example, the Massachusetts Institute of Technology (MIT) and makers of the App Private Kit have overcome Android and iOS interoperability issues for COVID-19 contact tracing to track people in close proximity with others using Bluetooth.^28^ The Bluetooth records when 2 devices running the App are near each other and notifications can be sent when people who crossed their path test positive for COVID-19. However, this requires use of a cell phone and download of an App. Creating privacy issues and obstacles. Mass gatherings are unique as they can overcome this privacy challenge by making a bracelet or similar device a condition of entry.

**FIGURE 1.**
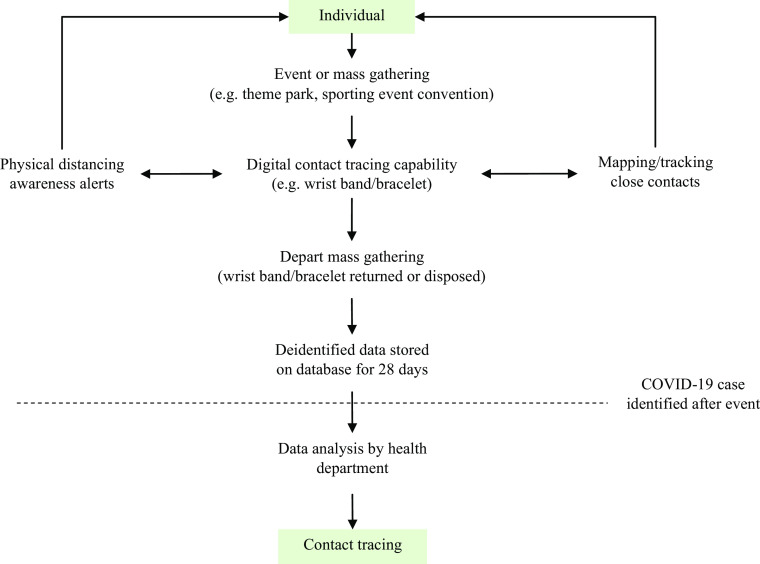
Concept for Digital Contact Tracing Solutions for Mass Gatherings

It is proposed that a Bluetooth or similar pairing device, worn as a wrist band/bracelet, lapel pin, or lanyard (among other options), be provided at mass gatherings to assess the nature of participant contacts (Figure 1). This simple approach is recommended due to the challenges associated with the use of other technologies that increase concerns related to privacy and trust. For example, people often do not want Apps installed on their phones and can be reluctant to use QR codes. It is true that participants would need to provide proof of identity and agree to wear the contact tracing devices for such a system to function effectively, but these prerequisites are both familiar to participants of many other activities or entry to facilities. Such devices need not have GPS or other position tracking capabilities; they only need to be able to pair with other participants devices when those devices come within 6 ft (2 m) of each other and time the duration of close contact.

Whenever close contacts occur, a “handshake” between the 2 is established and the elapsed time of contact is logged. Once a cumulative 15 min of contact within 6 ft occurs between any 2 participants, those individuals have established the CDC-designated criteria for transmission risk and the relationship is noted.^3^ Traditional tracing methods are generally ineffective at mass gatherings because many contacts occur between people who are not otherwise acquainted as a result of seating orientation and location or prolonged periods in close proximity in a line or near an attraction. Upon exiting the facility, the device (band/bracelet/lanyard/pin) would be deactivated and, depending on the type, returned or disposed. Recapture of the device may not be required to collect data, depending on the nature of the device.

The stored data on the wrist band/bracelet would be deidentified and held for 28 d. This would be achieved by using a specific code linked to the name, phone number, or email for each patron. In addition, the wrist band/bracelet could have the capability to buzz or shake if a person is within 6 ft of someone outside their party for 13 min. This would give a 2-min buffer for people to separate. The application of this could also act as a risk auditing measure by allowing mass gathering organizers to demonstrate measures in-place, compliance with CDC and other requirements, and identify choke points and other areas that may increase risk of exposure. In addition, this would strengthen the broader public health system by allowing other infectious disease contact tracing to be more efficient and accurate.

This approach can allow businesses, facilities, and event organizers to approach their local elected officials/health department to proactively seek permission to hold the event. Businesses would be able to demonstrate they have the mechanism required to develop a rapid plan of action for contact tracing. For example, if a person visiting the mass gathering tests positive to COVID-19 at a later date, the event organizer/company could provide the health department officials with information on movements and those who were in close contact. Ultimately, allowing contact tracers to sharpen their focus rather than a broad approach that may result in needing to contact thousands of people or put out a mass media program.

## IMPLEMENTATION

As business and government leaders develop strategies to reopen mass gatherings, the principles of intersectoral collaboration and risk management must be applied.^29-31^ If this does not occur, it will be very difficult for a coordinated and safe opening of mass gatherings. There are several important and even potentially “essential” gatherings, such as courtrooms, public hearings, schools, camps, homeless shelters, hospital, and high-density places of employment (meat processing) that will continue or commence shortly. These types of gatherings would also benefit from a wrist band/bracelet system once the mechanisms to facilitate use are developed. However, this can only be achieved by engaging with all sectors and agencies to consider the community wide benefits.

The contact tracing solution presented will require a shift in public thinking to go mainstream, which will include a social marketing campaign. This is why beginning with mass gatherings is the best first step to provide a vehicle for greater transition to societal pandemic resilience for the long term. In other words, this is more than just enabling mass gatherings, it is about allowing a faster total societal reopening. We have to strike now while there is a high-degree of value perceived in the reopening, which serves as the incentive for behavior change by wearing a Bluetooth or pairing type device while at a mass gathering.

Limitations may be raised during implementation. For example, will Bluetooth technology determine who is adjacent but separated by several people or an obstacle, reducing the risk profile. Although brief interactions are less likely to result in transmission, this system would not take into account the type of interaction (for example, did the infected person cough directly into the face of the exposed individual).^3^ This is an important factor but there is significant community information, guidance, signage, and awareness now of people to not attend locations if they have a fever, cough, or sore through. These issues along with other unknown challenges, not identified in this study, may occur. However, Bluetooth is a simple technology, established, and readily used. The premise of this solution is to enable rapid contact tracing of higher-risk interactions, overcoming privacy concerns, and facilitating resumption of mass gatherings.

Vital to implementation are policies that support focus on safety at mass gatherings and the ability to conduct rapid contact tracing. A key step for this is a strategy that streamlines processes and enhances contact tracing accuracy for event organizers and health department officials. In addition, the environmental health workforce should be enlisted to help guide and enforce sanitation, hygiene, and physical distancing requirements for mass gatherings. Achieving this will help reduce unemployment, enhance social protections and well-being, while bolstering business.^32^ Demonstrating a mature whole-of-society approach to COVID-19 recovery through mass gatherings while enhancing resilience for future pandemics and disease outbreaks.

## CONCLUSIONS

As communities begin initiating plans of incremental reopening, mass gatherings and high-density activities, such as sporting events, conventions, concerts, festivals, and theme parks, are consistently grouped among the highest-risk activities given the increased potential for widespread COVID-19 transmission. Absolute suppression of risk is unrealistic in all facets of life, but a more balanced risk management approach is suggested to enable safer resumption of activities that offer significant social and economic benefits. Contact tracing remains a limiting factor in achieving such a balance. The use of Bluetooth devices and advanced data analytics at mass gatherings is proposed to address this challenge. This simple approach is recommended due to the challenges associated with the use of technology that pertain to privacy and trust. Wearing the device would be a condition of entry. It would identify other participants that come within 6 ft (2 m) of another person for a cumulative 15 min of contact, the CDC-designated criteria for transmission risk. Upon exiting the facility, the device would be deactivated and, depending on the type of device, returned or disposed. If an attendee later tests positive for COVID-19 and tracing is required, the event organizer/company can provide a limited list of potential close contacts rather than an exhaustive list of all attendees. Contact tracers can, therefore, limit their efforts to this concise list rather than needing to contact thousands of people or conduct mass media communications. Such a system, if institutionalized, supports risk assurance and safety measures for businesses by demonstrating a commitment to staff, customer protection, and ensuring high-risk encounters are logged, reinforcing longer-term societal pandemic resilience.
